# Lateral patellar maltracking and patellar alta in the absence of increased TT-TG_dynamic_ distance in isolated patellofemoral pain: a dynamic kinematic CT study

**DOI:** 10.1186/s12880-025-01999-1

**Published:** 2025-11-05

**Authors:** Jia Li, Yurou Chen, Mao Yuan, Haitao Yang, Furong Lv, Bo Sheng, Wei Huang

**Affiliations:** 1 Department of Radiology, The First Affiliated Hospital of Chongqing Medical University, Chongqing, China; 2 Department of Orthopedic, The First Affiliated Hospital of Chongqing Medical University, 1 Youyi Road, Yuzhong Distract, Chongqing, 400016 P.R. China; 3 Orthopedic Research Laboratory, Chongqing Medical University, Chongqing, China

**Keywords:** Isolated patellofemoral pain, Dynamic kinematic CT, Patellar tracking, Lateral patellar maltracking, TT-TG distance

## Abstract

**Background:**

Patellar tracking in isolated patellofemoral pain needs further confirmation under dynamic conditions. The present study aimed to analyze the characteristics of patellar tracking and tibial tubercle-trochlear groove (TT-TG_dynamic_) distance during active knee motion in patients with isolated patellofemoral pain (PFP) via dynamic kinematic computed tomography (DKCT), and clarify whether the abnormal patellar tracking and TT-TG_dynamic_ distance are associated with isolated PFP.

**Methods:**

A total of 47 knees with isolated PFP and 47 control knees were scanned with DKCT. The TT-TG_dynamic_ distance, bisect offset (BO) index, lateral patellar tilt (LPT), and patellotrochlear index (PTI) were evaluated. All parameters were analyzed by two-factor repeated measures ANOVA.

**Results:**

In the two groups, the TT-TG_dynamic_ distance, BO index, LPT, and PTI varied with the knee flexion angle (all *p*<0.001). Between the two groups, the variation trends of TT-TG_dynamic_ distance and BO index were consistent (*p* = 0.131, 0.284, respectively), while those of LPT and PTI were significantly different (*p* = 0.001, <0.001, respectively). The BO index and LPT were significantly higher in the PFP group than those in the control group (*p* = 0.002, <0.001, respectively), and the PTI was significantly lower in the PFP group than that in the control group (*p*<0.001), whereas the TT-TG_dynamic_ distance showed no significant difference between the two groups (*p* = 0.631).

**Conclusions:**

Abnormal lateral patellar displacement, tilt and patellar alta occurred in the absence of increased TT-TG_dynamic_ distance in isolated PFP without trochlear dysplasia. Etiologic analysis of isolated PFP should emphasize lateral patellar tracking and patellar height.

## Introduction

Isolated patellofemoral pain (PFP) refers to the pain not secondary to patellar dislocations, osteoarthritis, or knee trauma [1, 2]. PFP is a common knee complaint in orthopedic practice, reducing quality of life and leading to long-term sequelae such as osteoarthritis [3–5]. Management of PFP remains challenging due to multifactorial etiology [6], and most patients with PFP reported persistent symptoms at long-term follow-up [7]. PFP does not seem to be self-limited, therefore, recognizing risk factors contributing to its development is essential to designing appropriate treatment plans and effectively improve symptoms. It was reported that abnormal patellar tilt and increased tibial tubercle-trochlear groove (TT-TG) distance are associated with PFP [8]. Yet some studies found that PFP patients are not necessarily associated with abnormal patellar tilt and increased TT-TG distance [9, 10]. In studies on patellar instability and patellar dislocation, the pathological patellofemoral alignment patterns result from multiple anatomical factors including increased TT-TG distance, trochlear dysplasia, torsional abnormality, and medial patellofemoral ligament insufficiency [11, 12]. However, the necessity of each factor is debated; notably, a subset of patients with patellar instability presents with normal TT-TG values, suggesting that other anatomical abnormalities alone can be sufficient to cause instability [13, 14]. Meanwhile, the potential value of other patellofemoral alignment parameters such as patellar tilt and shift should not be overlooked. Inspired by this, we became curious about the risk factor patterns for PFP especially isolated PFP. It is important to clarify that, compared to patients with patellar dislocation exhibiting obvious patellofemoral instability, isolated PFP presents with a more subtle and concealed form of patellar maltracking. It is insufficient to cause gross patellofemoral instability but sufficient to induce abnormal stress distribution on the articular surfaces, thereby triggering pain. Isolated PFP may be driven more by dynamic muscle imbalances and soft tissue constraints than by severe skeletal abnormalities. The primary focus of this study is precisely on patients with isolated PFP without severe objective instability. In addition, these studies were based on static examinations at a certain angle of the knee. PFP is the result of a combination of lower extremity malalignment and muscle imbalances around the knee during movements [15]. Static patellofemoral malalignment has a place in clinical practice. However, muscle activation during exercise can alter the static patellofemoral alignment identified in previous studies. This means that additional potential abnormalities may exist. Therefore, it is necessary to analyze the patellar tracking and the TT-TG_dynamic_ distance (TT-TG distance continuously measured at various angles of knee flexion-extension) during dynamic knee motion in patients with isolated PFP.

Many authors have advocated a dynamic perspective on the patellofemoral joint [6, 16–18]. Dynamic kinematic computed tomography (DKCT) is a new technology in musculoskeletal research for capturing continuous active patellar motion over a period of time [17, 19, 20]. Currently, the application of DKCT in the patellofemoral joint mainly focused on analyzing the characteristics of patellar tracking in patients with patellar dislocation [17, 20–22], and these studies have demonstrated that DKCT is an effective technique to analyze kinematic characteristics of the patellofemoral joint.

This study aimed to analyze the characteristics of patellar tracking and TT-TG_dynamic_ distance during active knee flexion and extension in patients with isolated PFP via DKCT, and clarify whether the abnormal patellar tracking and TT-TG_dynamic_ distance are associated with isolated PFP. We hypothesized that in patients with isolated PFP, abnormal lateral patellar displacement, tilt and patellar alta would occur in the absence of lateralization of tibial tuberosity.

## Materials and methods

### Participants

This study was approved by institutional review board approval (No. 2021 − 105) in accordance with the Declaration of Helsinki. This prospective cross-sectional study recruited PFP patients from January 2021 to June 2022 in the outpatient clinic of orthopedics. Two orthopedic surgeons performed the physical examination and clinical diagnosis for these patients. Inclusion criteria were as follows: (1) typical clinical signs and symptoms of PFP in the knee (typically presenting as diffuse pain behind or around the patella, and usually during activities such as jumping, squatting, running and up-down stair) [23]; (2) continuous presence of PFP symptoms in the knee for at least 6 months; and (3) magnetic resonance imaging (MRI) confirmed the absence of trochlear dysplasia (according to Dejour classification, sulcus angle, trochlear depth, and lateral trochlear inclination), ligament or meniscus injury. Exclusion criteria were as follows: (1) participants with traumatic onset of PFP; (2) affected knee with history of knee surgery; (3) previous patellar dislocation; (4) osteoarthritis (≥ Kellgren-Lawrence 3 grade); (5) incomplete imaging or poor imaging quality; (6) participants with contraindications to CT scan and (7) trochlear dysplasia.

We had recruited patients who required CT scans (e.g., injury, benign tumor, preoperative planning for anterior cruciate ligament repair surgery, etc.). The healthy side of these patients, as well as the affected side of patients with injury and benign tumor (excluding the anterior cruciate ligament tear patients) that had no involvement of the knee joint (distal tibia or proximal femur) were included in the control group. The inclusion criteria were no symptoms or discomfort in the patellofemoral joint. The patellofemoral and tibiofemoral functions were confirmed to be normal after physical examinations by orthopedic surgeons, and no meniscus or ligament injuries were confirmed by MRI. Exclusion criteria were consistent with those of the patient group.

DKCT data of examinations with poor image quality and captured maximum knee flexion angle less than 60° were excluded.

### Dynamic kinematic CT protocol

**Fig. 1:**
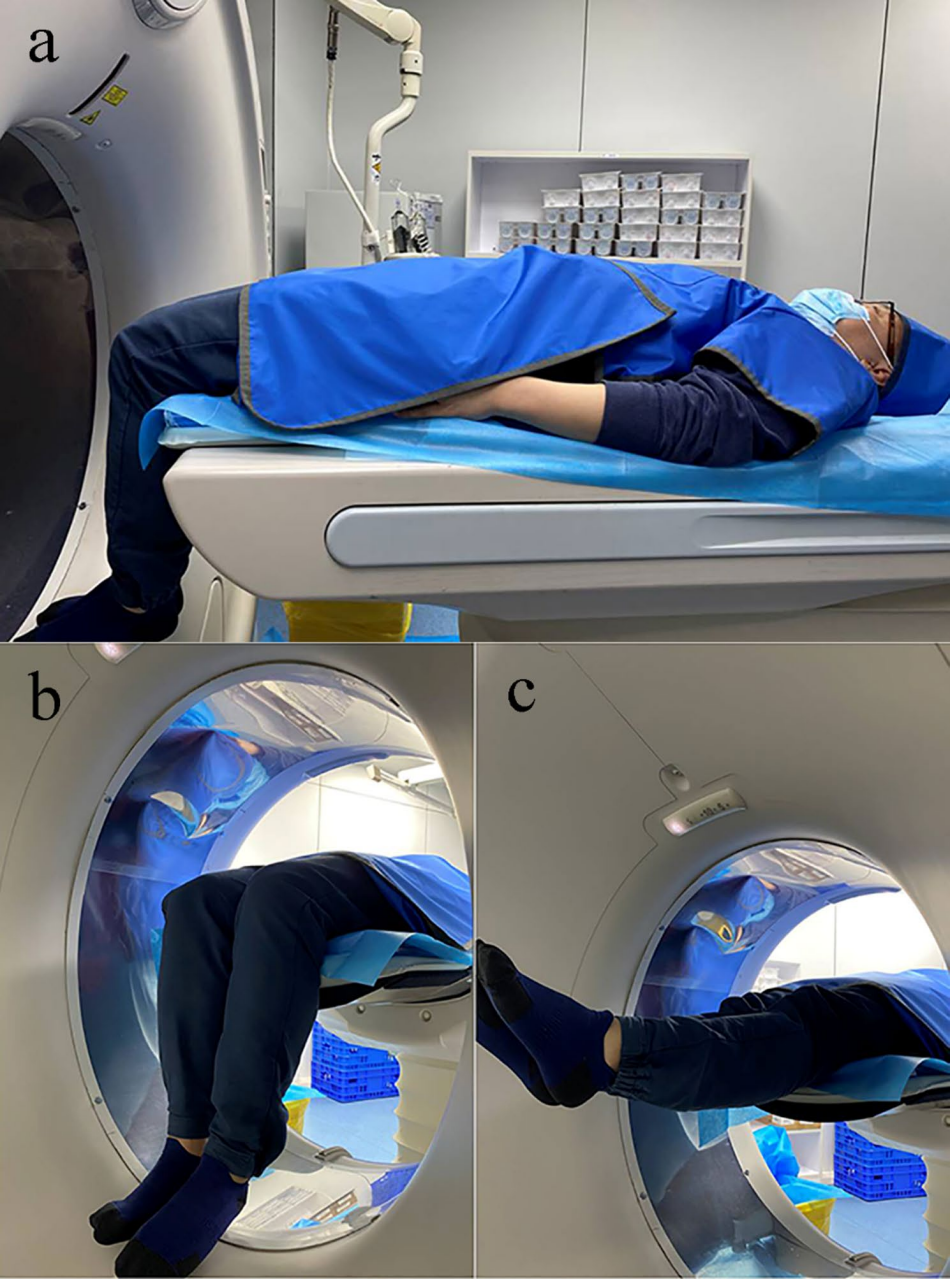
Position of participants during DKCT scans. (**a**) Participant is in supine position, with his/her head and neck to the proximal thigh covered by lead protector to reduce ionizing radiation in these areas; (**b**) and (**c**) DKCT scan of the knee in its maximum flexion phase could be achieved within the bore of the scanner and the knee at full extension

DKCT was performed on a wide detector CT scanner with 320 0.5-mm detectors (Aquilion ONE, Canon Medical Systems, Otawara, Japan). We used dynamic CT continuous scan mode (no table feed) to obtain three-dimensional kinematic imaging. All participants underwent standardized pre-scan training (≥ 5 practice trials) to achieve smooth and rhythmic motion. The participants were in supine position, with the thighs secured with a strap to keep the thighs in a fixed position, and the calves were placed outside the gantry and without restraint, so that the knees were free to flex and extend (Fig. 1). The participants completed approximately 1.5 cycles of knee motion (flexion-extension-flexion) at a constant speed during a 10-second scan time. The scanning protocol was as follows: slice thickness 0.5 mm, slice spacing 0.5 mm, tube rotation 0.35 s, tube output 100 kV and 70 mAs, time interval 0.5 s, DFOV 500 mm × 500 mm × 160 mm (LL). During the CT scan, the neck to the proximal thigh of each participant was covered with lead protector to reduce ionizing radiation in these areas. The effective dose for a single DKCT acquisition was 0.5 mSv and the CTDIvol was 16.4 mGy.

### Image analyses

**Fig. 2:**
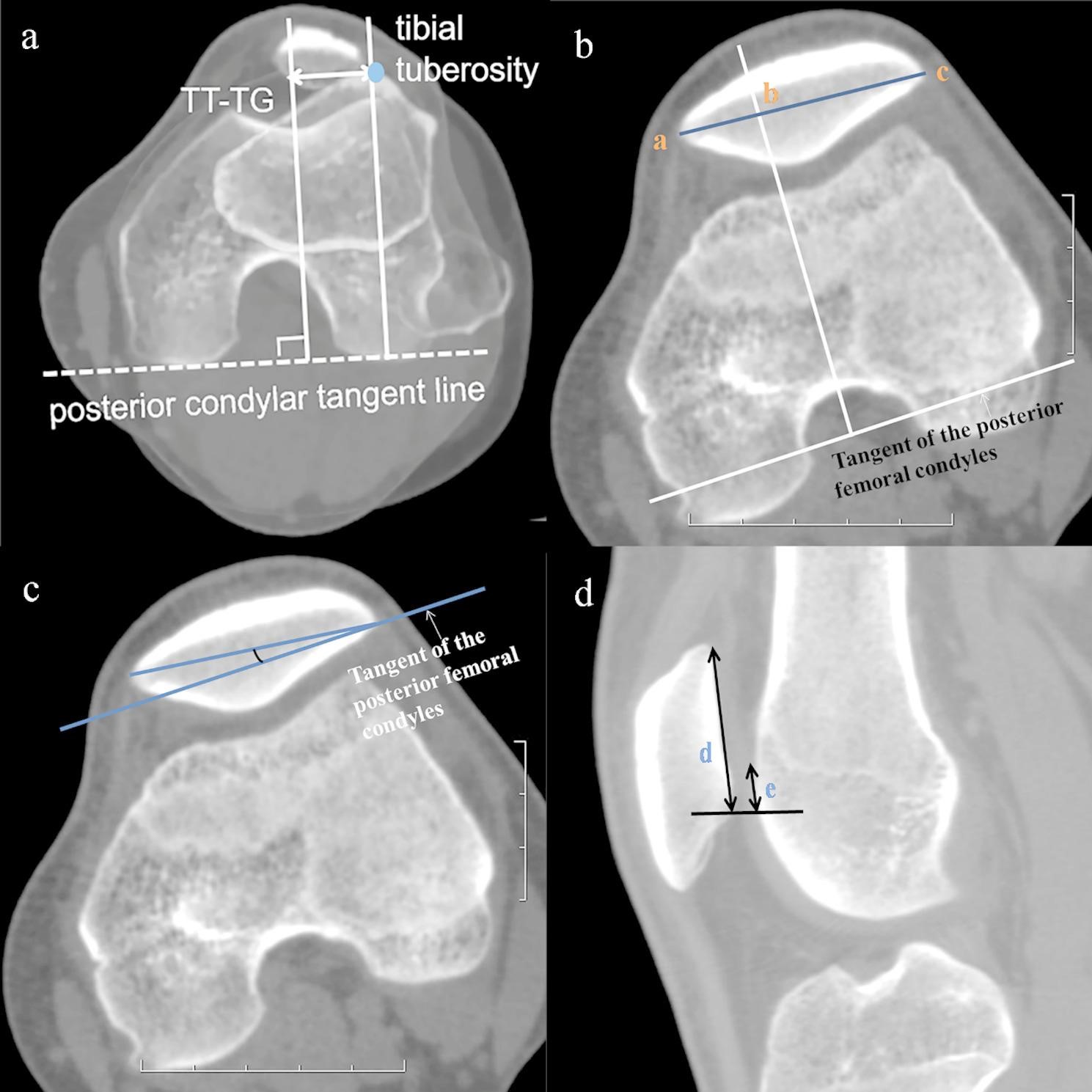
Methods for determining the TT-TG_dynamic_ distance, BO index, LPT and PTI. (**a**) The tibial tuberosity–trochlear groove_dynamic_ (TT-TG_dynamic_) distance is measured from the deepest point of the trochlear groove to the anterior-most point of the tibial tuberosity in line with the tangent of the posterior femoral condyles. (**b**) Bisect offset is measured as the percentage of the patellar axis (the line between the medial and lateral articular margins of the patella) to the trochlear line (bc/ac × 100). (**c**) The lateral patellar tilt (LPT) is defined as the angle between the patellar axis and the tangent line of the posterior femoral condyles. (**d**) The patellotrochlear index (PTI) is calculated as the distance between the most superior aspect of the trochlear articular surface to the most inferior aspect of the patellar articular cartilage (**e**) divided by the patellar articular surface length (**d**). The PTI is measured on the midsagittal section, which slice has the largest area of the patella

**Fig. 3:**
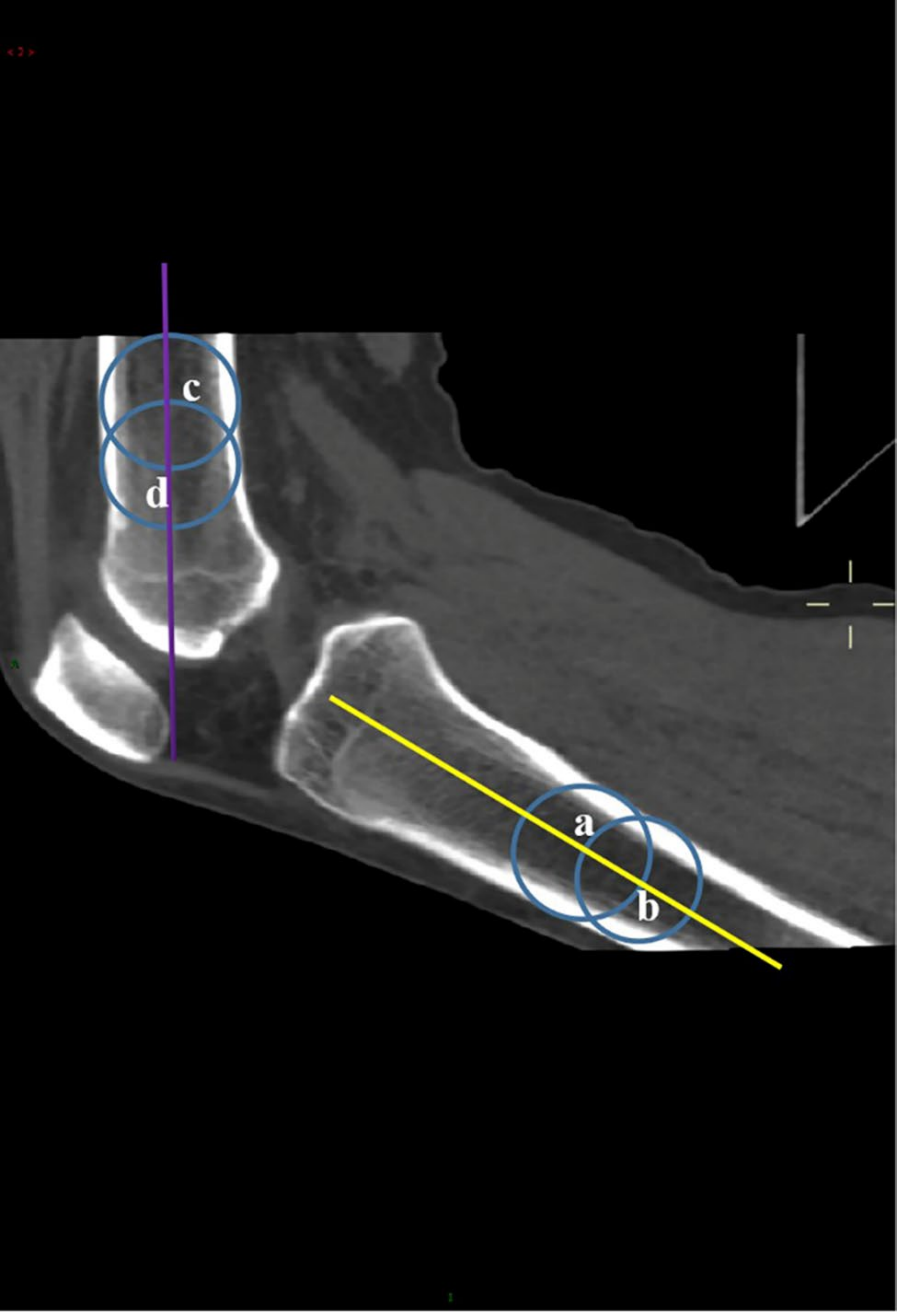
Method for measuring the flexion angle of the knee. Two circles were made on the median level of the tibia, and the edges of the circles intersected with the cortex of tibia and the center of the other circle. The line (solid yellow line) between the centers (**a, b**) of the two circles was the long axis of the tibia. In the same way, two circles were drawn on the femur. The line (purple solid line) between the centers (**c** and **d**) of the two circles was the long axis of the femur, and the angle between yellow and purple solid line was the knee flexion angle

In the study, we obtained three-dimensional CT images of the knee for 21 time frames during active extension and flexion. All parameters were measured on the images for each time frame. The TT-TG_dynamic_ distance [20] (Fig. 2a) was used to measure the lateralization of tibial tubercle with a cutoff of 20 mm [11], and bisect offset (BO) index [17] (Fig. 2b) was measured to evaluate the lateralization of patella with a cutoff of 66%. Patellar tilt was assessed by measuring the lateral patellar tilt (LPT) [20] (Fig. 2c) with a cutoff of 15°. Patellar height was measured using patellotrochlear index (PTI) [24] (Fig. 2d), which was performed in the sagittal plane, where the vertical length of the patella was the longest, with a cutoff of 0.28. The knee flexion angle was measured by drawing intersecting lines along the center of the femoral and tibial shafts (Fig. 3). Measurements were independently performed by two fellowship-trained musculoskeletal radiologists (Li and Chen, with 9 and 14 years of clinical experience respectively) after all the data from two groups were mixed, and randomly assigned in a blinded manner. All measurements were repeated by one of the radiologists (Li), and the interval time between the two measurements was at last four weeks.

### Statistical tests

The normality of continuous variables was verified by Shapiro-Wilk test, and *p* > 0.05 indicated that the data were in line with the normal distribution. Continuous variables with normal distribution are expressed as mean ± standard deviation. The independent sample t-test was used to compare the differences in age and BMI between the two groups. Chi-square test was used to compare the differences in gender and side between the two groups. Intra- and inter-observer reliability of quantitative measurements were assessed using intraclass correlation coefficients (ICCs), and a two-way random model [ICC (2,1)] was applied. ICC values were interpreted as follows: <0.50 (poor reliability), 0.50–0.75 (moderate reliability), >0.75 (good reliability), and >0.90 (excellent reliability) [25]. The differences between groups were analyzed using two-factor repeated measures analysis of variance. The main effects were group and knee flexion angle, and the interaction effect was used to assess the differences between groups in parameter change trends. If the data did not satisfy the spherical hypothesis, Greenhouse-Geisser correction was used. Bonferroni correction was used to further analyze the differences between groups at different flexion angles, and the significance level was set to α = 0.007 (0.05/7 angles). The above statistical analyses were performed using Windows software SPSS (version 17.0; SPSS, Chicago, IL, USA), and the significance level was set at 0.05. GPower software (version 3.1.9.6) was used to perform calculation on sample size. The effect size, minimal significance (alpha) and statistical power (1 - beta) were set at 0.25, 0.05 and 0.80 respectively [26], and the minimum sample size was 68 cases.

## Results

### Demographic characteristics of the subjects in the two groups

During the study period, 62 knees with PFP underwent DKCT scans, 8 knees were excluded due to poor image quality, and 7 knees were excluded due to the presence of severe osteoarthritis. Finally, a total of 47 knees from 28 patients (8 males, 20 females) were included in the PFP group. 55 control knees completed DKCT scans, 4 knees were excluded due to poor image quality, and 4 knees were excluded due to the maximum knee flexion angle of less than 60°. At last, there were 47 knees from 41 subjects (29 males, 12 females) in the control group. There was no difference between PFP and control groups in terms of side. Although the age of the PFP group was higher than that of the control group (*P* = 0.047), the vast majority of both groups were at or at least near skeletal maturity. We are unware of any evidence to suggest that the anatomic factors measured in the present study change over time after skeletal maturity [27]. There was difference in gender between PFP and control groups (*P* <0.001), but heterogeneity test showed that there was no heterogeneity between male and female. There was also difference in BMI between PFP and control groups (*P* <0.001). Due to difficulties in recruiting subjects caused by radiation concerns related to DKCT, we were unable to match the BMI between the two groups (Table 1).

**Table 1:** Participant characteristics

Characteristic	PFP group (*n* = 47)	Control group (*n* = 47)	*P* value
Gender (females/males)	20/8	12/29	**<0.001**
Side (right/left)	26/21	19/28	0.148
Average age, y	32 ± 7	28 ± 7	**0.047**
Age range, y	17–44	17–43	NA
BMI (body mass index), kg/m^2^	21.60 ± 2.13	23.36 ± 1.89	**<0.001**
VAS pain scores (out of 10)			
Pain during a typical day	2.48 ± 0.82	—	NA
Pain during a provocative activity	4.69 ± 0.85	—	NA

### Intra- and inter-observer reliability of quantitative measurements

As shown in Table 2, the intra-reader ICCs for the four quantitative measurements ranged from 0.867 to 0.985, and the inter-reader ICCs ranged from 0.808 to 0.969, indicating good to excellent intra- and inter-reader reliability.

**Table 2:** The intra- and inter-observer agreement of quantitative measurements between two radiologists

Measurement	Intra-observer ICC(95% CI)	Inter-observer ICC(95% CI)
TT-TG_dynamic_ distance	0.958(0.897, 0.983)	0.912(0.792, 0.964)
BO index	0.880(0.723, 0.951)	0.852(0.664, 0.939)
LPT	0.867(0.698, 0.945)	0.808(0.583, 0.919)
PTI	0.936(0.849, 0.974)	0.969(0.922, 0.987)

*TT-TG*_*dynamic*_ *distance*: the tibial tubercle–trochlear groove_dynamic_ distance

*BO index*: bisect offset index

*LPT*: lateral patellar tilt

*PTI*: patellotrochlear index

*ICCs*: intraclass correlation coefficients

*CI*: confidence interval

### Comparison of the four parameters between the PFP group and control group

The TT-TG_dynamic_ distance gradually decreased as the knee flexion angle increased from − 10° to 60°, (F(6, 87) = 65.116, *p*<0.001). And the variation trend was consistent (F(6, 87) = 1.980, *p* = 0.131). There was no statistically significant difference in TT-TG_dynamic_ distance between the two groups (F(1, 92) = 0.233, *p* = 0.631). The differences in TT-TG_dynamic_ distance measurements between the two groups at different flexion angles were further compared, and there was no statistical difference in TT-TG_dynamic_ distance between the two groups at all flexion angles (all *p* > 0.007).

As the knee flexion angle increased, the BO index gradually decreased and then increased (F(6, 87) = 21.842, *p*<0.001). And the variation trend was consistent between the two groups (F(6, 87) = 1.260, *p* = 0.284). The BO index in the PFP group was significantly higher than that in the control group (F(1, 92) = 10.663, *p* = 0.002). The difference in BO index between the two groups under different flexion angles was further compared. The BO index in the PFP group was significantly higher than that in the control group when the knee joint was at-10°-0° and 0°-10°(F(1, 46) = 13.210, *p* = 0.001), (F(1, 46) = 10.573, *p* = 0.002), respectively.

The LPT varied with the knee flexion angle (F(6, 87) = 14.654, *p*<0.001), and the variation trend was significantly different between the two groups (F(6, 87) = 4.158, *p* = 0.001). The LPT in the PFP group was significantly higher than that in the control group (F(1, 92) = 21.800, *p*<0.001). The differences in LPT between the two groups under different flexion angles were further compared. The LPT in the PFP group were significantly higher than those in the control group in all knee flexion angle (all *p* < 0.007).

**Fig. 4:**
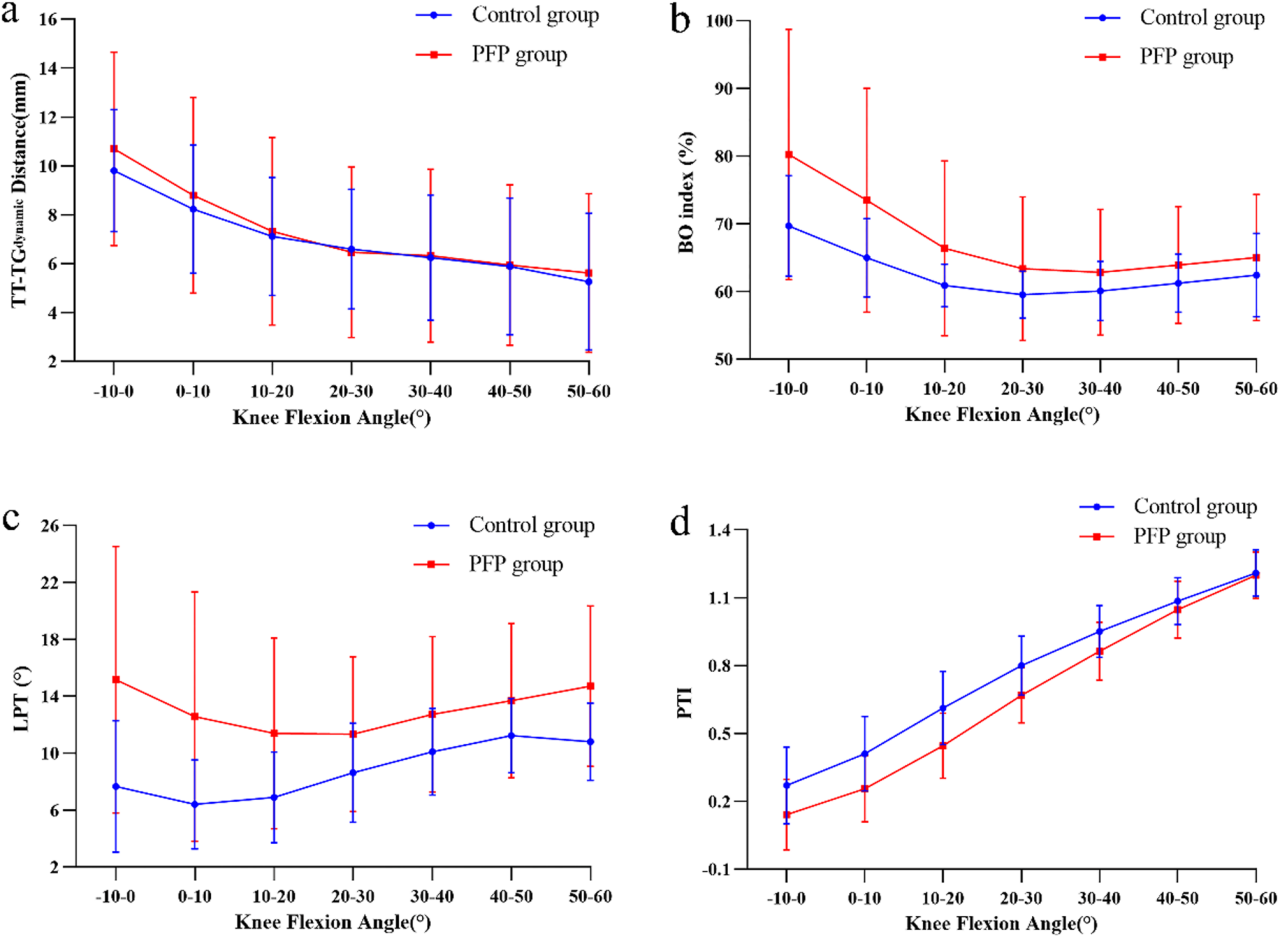
Mean (± standard deviation) interpolated data for the (**a**) TT-TG_dynamic_ distance; (**b**) BO index; (**c**) LPT; and (**d**) PTI versus knee flexion angle

The PTI gradually increased when the knee flexion angle increased from − 10° to 60° (F(6, 87) = 804.701, *p*<0.001), with a significant difference in the variation trend between the two groups (F(6, 87) = 8.193, *p*<0.001). PTI in the PFP group was significantly lower than that in the control group (F(1, 92) = 19.623, *p*<0.001). The differences in PTI between the two groups under different flexion angles were further compared. When the knee flexion angle was-10°-40°, the PTI in the PFP group was significantly lower than that in the control group (all *p* < 0.007) (Table 3; Fig. 4).

**Table 3:** Comparison of the TT-TG Distance, BO Index, LPT and PTI between PFP group and control group^#^

Knee Flexion Angle (°)	TT-TG Distance (mm)		BO Index (%)		LPT (°)		PTI	
	PFP group	Control group	PFP group	Control group	PFP group	Control group	PFP group	Control group
–10 − 0	10.7 ± 4.0	9.8 ± 2.5	80.3 ± 18.5	69.7 ± 7.4	15.2 ± 9.4	7.7 ± 4.6	0.1 ± 0.2	0.3 ± 0.2
0–10	8.8 ± 4.0	8.2 ± 2.6	73.5 ± 16.6	65.0 ± 5.8	12.6 ± 8.8	6.4 ± 3.13	0.3 ± 0.1	0.4 ± 0.2
10–20	7.3 ± 3.8	7.1 ± 2.4	66.4 ± 12.9	60.9 ± 3.2	11.4 ± 6.7	6.9 ± 3.2	0.4 ± 0.1	0.6 ± 0.2
20–30	6.5 ± 3.5	6.6 ± 2.4	63.4 ± 10.6	59.5 ± 3.5	11.3 ± 5.4	8.6 ± 3.5	0.7 ± 0.1	0.8 ± 0.1
30–40	6.3 ± 3.5	6.2 ± 2.6	62.9 ± 9.3	60.1 ± 4.4	12.7 ± 5.5	10.1 ± 3.0	0.9 ± 0.1	1.0 ± 0.1
40–50	6.0 ± 3.3	5.9 ± 2.8	63.9 ± 8.6	61.2 ± 4.3	13.7 ± 5.4	11.2 ± 2.6	1.0 ± 0.1	1.1 ± 0.1
50–60	5.6 ± 3.2	5.3 ± 2.8	65.0 ± 9.3	62.4 ± 6.2	14.7 ± 5.7	10.8 ± 2.7	1.2 ± 0.1	1.2 ± 0.1

^**#**^: The *p* value of BO index, LPT, PTI, and TT-TG distance were 0.002, <0.001, <0.001, 0.631, respectively (i.e., overall differences between groups)

PFP: patellofemoral pain

*TT-TG distance*: the tibial tubercle–trochlear groove distance

*BO index*: bisect offset index

*LPT*: lateral patellar tilt

*PTI*: patellotrochlear index

## Discussion

The most important finding of this study was that abnormal lateral patellar displacement, tilt and patellar alta occurred in the absence of increased TT-TG_dynamic_ distance in isolated PFP. This finding supported our hypothesis.

As early as more than 20 years ago, researchers have proposed the need to evaluate the characteristics of patellofemoral alignment from a dynamic perspective [28]. A study by Best MJ et al. [22] mentioned the use of visual assessment alone to detect the presence or grade of patellar maltracking was not reliable. The evaluation of patellar tracking needs some more objective examination methods. Advantages of DKCT include allowing active joint movement during scanning, images with high temporal resolution and limited movement artifacts [19]. Our results also supported DKCT as an effective technique for analyzing the kinematics pattern of the patellofemoral joint.

This study analyzed the changes in patellofemoral alignment during knee motion. Results showed that TT-TG_dynamic_ distance, BO index and LPT were higher in the extended knee and decreased with knee flexion, which was consistent with the results of Williams AA et al. [21]regarding the evaluation of patellofemoral kinematics characteristics in patients with unilateral patellofemoral instability. The tibia rotates externally related to the femur during knee extension, and tibial tuberosity lateralization increases the lateralizing force exerted by the patellar tendon on patella [21, 29, 30]. However, the lateralizing force of patellar tendon is counteracted by the femoral trochlea during knee flexion, which brings the patella to the more centralized tracking [21].

Interestingly, between the two groups, there was no significant difference in TT-TG_dynamic_ distance and the variation trend of TT-TG_dynamic_ distance during knee motion was consistent, which was different from previous studies. We analyzed that this might be due to the fact that subjects of these previous research were mainly patients with patellar dislocation [20, 21, 31, 32], and analysis were based on static examinations or MRI [33, 34]. In our study, to minimize the influence of patellofemoral morphology on patellar tracking, we excluded patients with trochlear dysplasia and history of patellar dislocation. The results of our study suggested that the location of tibial tuberosity might be normal in isolated PFP patients without severe trochlear deformity and history of patellar dislocation, which might be the reason for the medial patellar instability and suboptimal prognosis after tibial tuberosity osteotomy in patients with PFP reported in some studies [35, 36]. Caution is needed when considering tibial tuberosity osteotomy for isolated PFP. Several studies have reported poor outcomes after tibial tuberosity osteotomy for isolated PFP [37, 38]. We assumed that such poor postoperative outcome was due to overcorrection caused by inaccurate and incomplete preoperative evaluation of the TT-TG distance. Therefore, in patients with isolated PFP who failed conservative treatments, dynamic examinations should be performed to assess the TT-TG_dynamic_ distance in the functional state of the knee.

Numerous studies have confirmed that assessing lateral patellar shift and tilt during activities requiring active quadriceps can improve the ability to diagnose patellar maltracking [1]. The results of our study were consistent with previous studies that lateral patellar shift and tilt were higher in the PFP group than in the control group [33, 39]. Our data showed that the lateral patellar displacement and tilt were better indicators than TT-TG_dynamic_ distance in patients with isolated PFP. In addition, the present research found that the lateral patellar tilt pattern during active knee flexion and extension was different between the two groups. This result further emphasized the role of imbalanced muscle force in the development of PFP and the importance of evaluating patellofemoral kinematics under active quadriceps conditions [1, 40–43].

In the present study, we used PTI as an indicator to evaluate patellar height because some studies suggested that PTI could be better used to assess the vertical position of the patella [24, 44]. Our data showed that the PFP group has a higher patella position than the control group, and there was an abnormal pattern of patella height change in the PFP group during active knee flexion and extension. Several authors have found that patellar alta was an important and common anatomical factor associated with patellar instability [45, 46]. A higher patella may lead to excessive lateral motion of the patella during knee flexion and cause the patella to fail to engage the trochlear groove early during knee flexion [47]. To the best of our knowledge, this study was the first to analyze the pattern of PTI changes during active knee flexion and extension. The effectiveness and clinical practical value of abnormal patella height change patterns in the development of PFP requires more in-depth research.

Our study had several limitations. First, there were significant differences between the two groups in terms of gender, age, and BMI, and these demographic factors might be confounding factors. The vast majority of both groups were at or at least near skeletal maturity. We are unware of any evidence to suggest that the anatomic factors measured in the present study change over time after skeletal maturity. Heterogeneity test showed that there was no heterogeneity between male and female. It is true that BMI between the two groups needs to be strictly matched. Due to difficulties in recruiting subjects caused by radiation concerns related to DKCT, we were unable to match the BMI between the two groups. And in future studies, we will strictly match the BMI of the two groups to eliminate the influence. Second, the parameters in this study were measured manually, and the value of these parameters needs to be validated by relevant biomechanical studies. Third, the control group included knees with lower extremity injuries or benign tumors (distal tibia or proximal femur) but unaffected patellofemoral function, in addition to normal knees. Heterogeneity analysis showed no heterogeneity between subgroups (the normal knee group and the affected but uninvolved knee group) of controls. Fourth, this study used the PTI to assess patellar height. PTI is a parameter measured based on MRI; however, validation comparing PTI measured on CT with that measured on MRI demonstrated excellent correlation. Therefore, while PTI was originally defined based on MRI measurement, it can also be applied to CT measurement. Fifth, the equipment used in this study can only achieve no-load active motion in the supine position. Ideally, dynamic scanning should be performed under weight-bearing conditions to better simulate the actual motion. Finally, the knee flexion angle was measured using a 2D method based on previous literature [48]. This method does not account for errors introduced by limb positioning, such as misalignment with the CT scanning axis. We would use 3D approach in future research to enhance precision.

## Conclusions

Abnormal lateral patellar displacement, tilt and patellar alta occurred in the absence of increased TT-TG_dynamic_ distance in isolated PFP without trochlear dysplasia. Etiologic analysis of isolated PFP should emphasize lateral patellar tracking and patellar height.

## Data Availability

The datasets used and/or analysed during the current study are available from the corresponding author on reasonable request.

## Abbreviations

BO: Bisect offset
CI: Confidence interval
DKCT: Dynamic kinematic computed tomography
ICCs: Intraclass correlation coefficients
LPT: Lateral patellar tilt
MRI: Magnetic resonance imaging
PFP: Patellofemoral pain
PTI: Patellotrochlear index
TT-TG: Tibial tubercle-trochlear groove
